# A twenty-year journey exploring coumarin-based derivatives as bioactive molecules

**DOI:** 10.3389/fchem.2022.1002547

**Published:** 2022-10-10

**Authors:** Leonardo Pisani, Marco Catto, Giovanni Muncipinto, Orazio Nicolotti, Antonio Carrieri, Mariagrazia Rullo, Angela Stefanachi, Francesco Leonetti, Cosimo Altomare

**Affiliations:** ^1^ Department of Pharmacy-Pharmaceutical Sciences, University of Bari Aldo Moro, Bari, Italy; ^2^ Photys Therapeutics, Cambridge, MA, United States

**Keywords:** cholinesterases, monoamine oxidases, coumarin, neurodegeneration, cancer

## Abstract

The coumarin core (i.e., 1-benzopyran-2 (2*H*)-one) is a structural motif highly recurrent in both natural products and bioactive molecules. Indeed, depending on the substituents and branching positions around the byciclic core, coumarin-containing compounds have shown diverse pharmacological activities, ranging from anticoagulant activities to anti-inflammatory, antimicrobial, anti-HIV and antitumor effects. In this survey, we have reported the main scientific results of the 20-years investigation on the coumarin core, exploited by the research group headed by Prof. Angelo Carotti (Bari, Italy) either as a scaffold or a pharmacophore moiety in designing novel biologically active small molecules.

## Introduction

Due to its occurrence in both natural products and drugs, and the synthetic accessibility of its derivatives as well, the coumarin core (i.e., 1-benzopyran-2 (2*H*)-one) has been considered a privileged scaffold for drug design. Coumarin was isolated from tonka bean by Vogel in 1820. After its structural elucidation, this chemical scaffold was found in many bioactive natural compounds. Noteworthy, the anticoagulants warfarin, the choleretics armillarisin A and the antibiotic novobiocin must be mentioned as marketed drugs based on the coumarin scaffold. However, coumarin based derivatives are not devoid of drawbacks concerning with solubility and multidrug resistance (Wu et al., 2020; Al-Abass et al., 2021) and this makes the design even more challenging. Indeed, depending upon feature and substitution pattern of the functional groups, the coumarin nucleus has been widely decorated to develop compounds showing diverse pharmacological activities, spanning from anticoagulant activities to anti-inflammatory, antimicrobial, anti-HIV and antitumor effects (Stefanachi et al., 2018).

Herein, we review the scientific results in the last 2 decades of investigation around the coumarin nucleus, developed in the research group of Prof. Angelo Carotti (Bari, Italy).

The interest toward coumarin derivatives by the Carotti’s group began in the early 2000 s from a project aimed at generating ligand-based models of neurotoxicity of MAO substrates, using the classical Hansch quantitative structure-activity relationships (QSARs) and comparative molecular field analysis (CoMFA) (Youdim et al., 2006). In details, the QSAR/CoMFA analyses focused on the MAO-catalyzed oxidation of 1-methyl-4-phenyl-1,2,3,6-tetrahydropyridine (MPTP) to 1-methyl-4-phenylpyridinium (MPP^+^), which produces Parkinson-like symptoms in primates (Altomare et al., 1992). The toxication of MPTP involves a two-step oxidation into the brain by MAO B, and to a lesser extent by MAO A. These CoMFA studies, combined with the Hansch 2D-QSAR analyses, allowed to spot the main steric effects, along with the electrostatic and hydrophobic interactions, most discriminating the binding sites in the two isozymes.

Besides the 3D QSAR studies on MPTP analogs, Carotti and coworkers investigated a number of fused azaheterocycles, like 5*H*-indeno [1,2-*c*] pyridazines and other condensed pyridazines and pyrimidines (Altomare et al., 1991), as MAO inhibitors, and derived QSAR and CoMFA models which highlighted key physicochemical interactions responsible for inhibition potency and A/B isoform selectivity (Kneubühler et al., 1993; Kneubühler et al., 1995) and, among them, coumarins showed really interesting results (Altomare et al., 1998; Gnerre et al., 2000)

Indeed, Carotti’s synthetic efforts towards several coumarin derivatives, along with 3D QSAR models, helped to unravel the key molecular features responsible for binding affinity to MAO A/B, inhibition potency and isoform selectivity (Altomare et al., 1998; Gnerre et al., 2000), even before that the co-crystal structures of MAOs with small molecule binders (Binda et al., 2007) opened the way to structure-based drug design of novel inhibitors. Since then, as discussed below, many of the group’s publications focused on potential applications of diverse coumarin-containing compounds in neurodegeneration and oncology.

## Coumarins as inhibitors of MAOs

**FIGURE 1:**
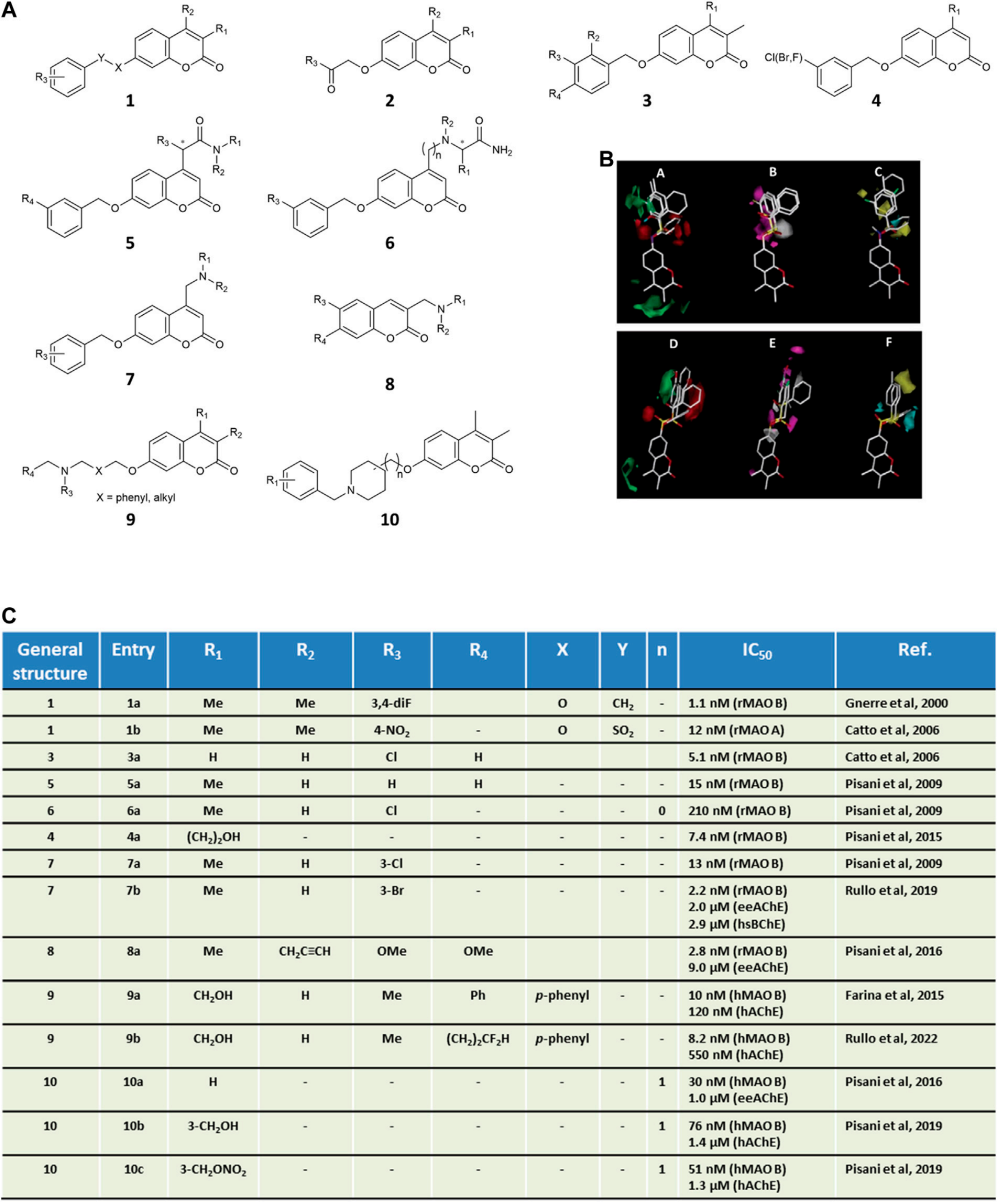
Panel **(A)** general structures of coumarin-based MAO inhibitors and MTDLs. Panel **(B)** CoMFA isocontour maps for MAO B **(A–C)** and MAO A **(D–F)** affinity of coumarin derivatives of general structure 1. In steric maps A and D, green and red regions show allowed and forbidden regions, respectively. Electronic density areas **(B,E)** are depicted as favorable (magenta) and unfavorable (grey), respectively. Lipophilic interactions **(C,F)** are reported as favorable (yellow) and unfavorable (cyan), respectively. CoMFA images were adapted with permission from Ref. (Catto et al., 2006). Copyright 2006, American Chemical Society. Panel **(C)** *in vitro* inhibitory data for the selected coumarins.

Due to higher synthetic feasibility, seminal explorations focusing on MAO inhibitors mainly addressed the coumarin substituent at the 7-position, while limiting the structural variations at position 3 and 4 to methyl groups (Figure 1, general structures **1**–**2**). The modulation of MAO B activity was linearly correlated to the substituent’s lipophilicity at C7 (Carotti et al., 2006). Different linkers were installed at C7, probing both size and electronic features, with the OCH_2_Ph group being the most effective in promoting MAO B inhibitory activity. Among 7-benzyloxy derivatives (Figure 1, general structure **3**), activity was highly dependent on the phenyl *meta*-substituent, whose lipophilicity governed MAO B inhibitory potencies rather than electronic properties, as outlined by a reliable QSAR model showing a parabolic correlation of potency with the hydrophobic Hansch constant π (Gnerre et al., 2000). These findings were later confirmed by disclosing a linear correlation between MAO B potency and calculated partition coefficient (ClogP) from a data set of coumarin derivatives exploring even more diverse substituents (De Colibus et al., 2005). A more extensive CoMFA-GOLPE study encompassing larger substitution patterns at the 7-position of the coumarin allowed to derive easily interpretable isocontour maps for both MAO A and MAO B affinity (Catto et al., 2006) (Figure 1). All steric, lipophilic, and electrostatic potential maps were found to slightly differ in the two isozymes, with some common features (e.g., a detrimental effect of *ortho* substitution). Noteworthy, CoMFA maps were successfully applied to shed light on MAO B/A selectivity, which was mainly modulated by the electronic and steric properties of the bridge connecting the coumarin core to the aryl substituent. Interestingly, the insertion of 7-phenylsulfonate esters reversed the selectivity profile yielding potent MAO A inhibitors (Catto et al., 2006). Complex stabilization was strongly promoted by sulfonate-group hydrogen bonding to the sidechain of Gln215 as supported by both molecular dynamics and molecular mechanics generalized Born surface area (MM-GBSA) calculations (Mangiatordi et al., 2017). With the aim of improving the drug-like properties of neutral coumarin hits, differently sized and substituted polar and/or protonatable moieties were introduced at the 4-position of 7-benzyloxy derivatives (Figure 1, general structure **5**–**6**) leading to the discovery of NW-1772 (Pisani et al., 2009), an *in vivo* potent, bioavailable, reversible and CNS-acting MAO B inhibitor devoid of safety issues. X-ray crystallography pointed out its binding within the MAO enzymatic cleft, fully buried from the entrance cavity to FAD in a safinamide-superimposable pose (Binda et al., 2007). While maintaining a *meta*-halobenzyloxy group at C7 (Figure 1, general structure **4**), fine-tuning the substituent at the 4-position returned nanomolar inhibitors that laid the groundwork for developing a robust and highly predictive Gaussian field-based 3D-QSAR model (Pisani et al., 2015), indicating that target recognition was dominated by steric hindrance whereas hydrogen bonding and electrostatic interactions tempered affinity to a lesser extent. During our investigation, serendipitously, while reacting 4-chloromethyl-7-substituted coumarins with excess primary amines, we faced an unexpected lactone opening reaction followed by intramolecular nucleophilic substitution (Pisani et al., 2013). This mechanism gave straightforward and efficient access to a novel scaffold for MAO inhibition, namely 6′-substituted (*E*)-2-(benzofuran-3 (2H)-ylidene)-*N*-alkylacetamides. Depending on the linker, sulfonate and benzyl derivatives confirmed A/B selectivity profiles obtained for the coumarin isomers.

## Coumarins as inhibitors of Acetylcholinesterase

**FIGURE 2:**
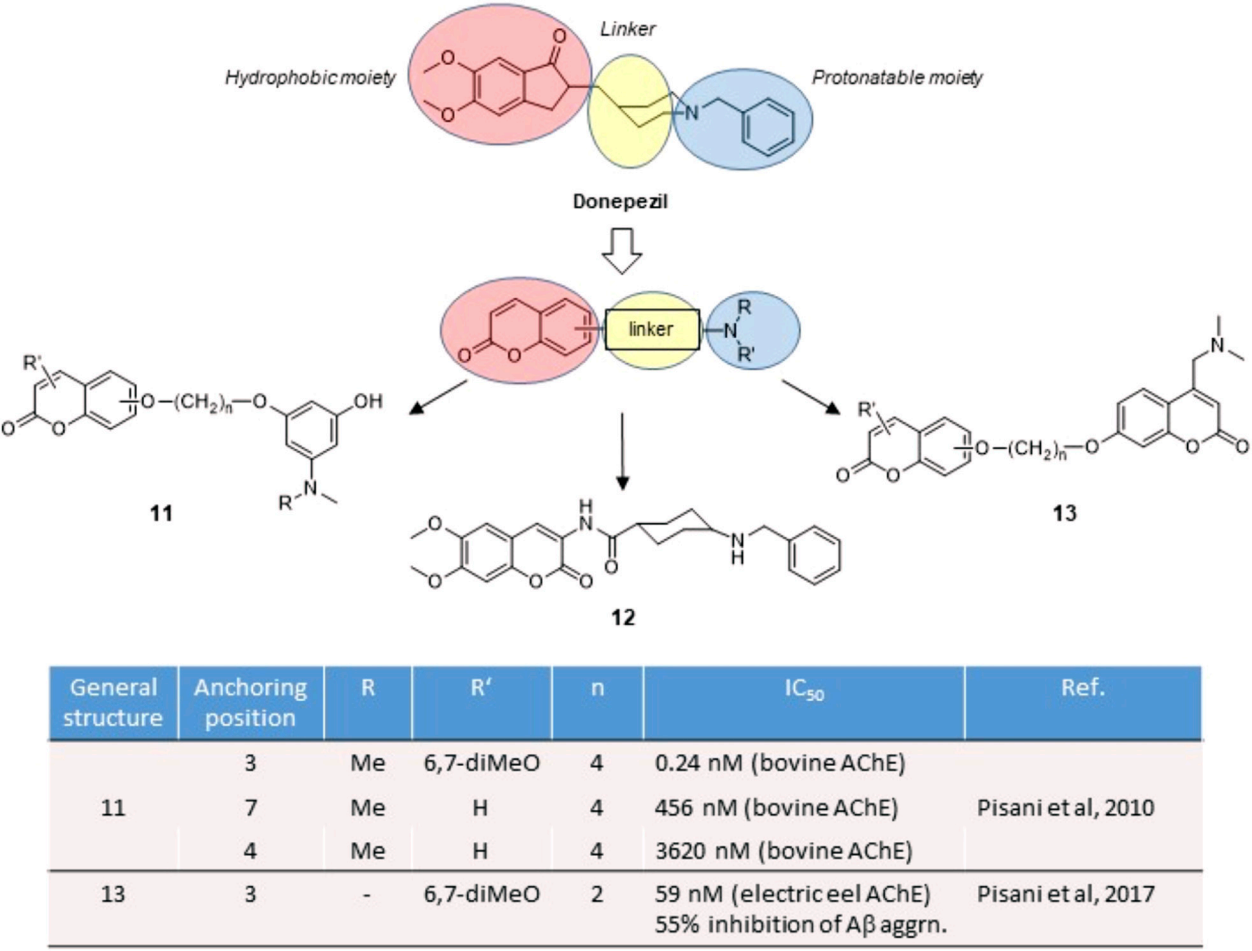
Rational design of AChE inhibitors by conjugative approach.

Acetylcholinesterase (AChE) is the enzyme responsible for the hydrolysis of the neurotransmitter acetylcholine (ACh) at the synaptic cleft. Because of many physiological effects mediated both in central and peripheral tissues, AChE is by far a validated pharmacological target. AChE inhibitors (AChEIs) are currently the first-line drugs for the symptomatic treatment of Alzheimer’s disease (AD), where AChEIs may sustain the cholinergic transmission in early to moderate AD stages (Mufson et al., 2008). The early crystal structures of AChE-inhibitor complexes revealed the presence of two binding pockets, a catalytic active site (CAS), surrounded by aromatic amino acids and neighbouring the so-called catalytic triad, and a peripheral binding site (PAS), separated by a deep gorge (Sussman et al., 1991). The presence of ammonium cationic warheads warrants the tight binding with both CAS and PAS, by means of cation-π interactions, as suggested by decamethonium and other neuromuscular blockers. To limit the peripheral effects of AChEIs and obtain CNS-active drugs, a protonatable nitrogen usually replaces the net positive charge of the ammonium ions for binding at the CAS, while a lipophilic moiety brought as the distal substituent acts as a second pharmacophore motif for π-π interactions with aromatic amino acids at the PAS. Such archetypal dual binding site (DBS) feature is represented by donepezil (Figure 2), the reference drug for the treatment of AD. Since the PAS is involved in the aggregation of beta-amyloid (Aβ) protein (Inestrosa et al., 1996), DBS inhibitors may exert antiamyloidogenic activity by disrupting protein-protein interactions triggered at the PAS.

Starting from the disclosure of a fair AChE inhibitory activity for the 7-benzyloxy coumarin derivatives (Brühlmann et al., 2001), our group focused on the exploitation of the synthetic versatility of the coumarin scaffold to obtain DBS reversible inhibitors. By conjugating in a fragment-based design an edrophonium-like moiety with hydroxycoumarin through a polymethylene bridge, we obtained chimeric derivatives **11** (Figure 2) (Leonetti et al., 2008; Pisani et al., 2010), with good IC_50_ values in the submicromolar range, and in some cases hitting subnanomolar values. Docking studies evidenced the interaction of the coumarin residue at the PAS through hydrophobic and π-stacking interactions and of the cationic head at the CAS as the key interactions for binding. A systematic exploration of the substitution pattern around the coumarin core outlined the best solutions for its decoration. An “extended” substitution in 3-, 6- and 7-position of the coumarin led to higher potencies than in case of “folded” substitution in 4-, 5- and 8-position.

The same activity trend was later confirmed by coumarins **12** and **13** (Figure 2), where the putative toxicophore fragment represented by the hydroxyaniline moiety was replaced with a simple benzylamine substituent or a protonatable coumarin building block. Such substitution, still matching the donepezil-like pharmacophore, retained an effective binding at both the CAS and the PAS (Catto et al., 2013), and was later confirmed by X-ray analysis of AChE in complex with inhibitor **12** (Catto et al., 2020). By following a conjugation approach, we disclosed a series of homo- and heterodimers **13** (Figure 2) (Pisani et al., 2017) where at least one coumarin residue presented a protonatable nitrogen as the key substituent. Moreover, the DBS interaction of these coumarin hybrids was accompanied with a direct antiaggregating activity against Aβ self-aggregation, thus representing an appealing example of multitarget activity directly related to AChE inhibition.

## Coumarin-based multitarget-directed ligands

Our contribution to the field of multi-targeting ligands has long been devoted to the development of dual coumarin-based AChE-MAO B inhibitors as potential polypharmacological weapons to combat AD. Apart from the well-established role of cholinergic transmission in AD, the rationale lies in the synergistic or additive effect coming from the oxidative stress restriction as a consequence of MAO blockade. In a seminal paper by some of us, the *in vitro* screening enrolling a small subset of 7-benzyloxycoumarins (Figure 1, general structure **3**), previously characterized as nanomolar MAO inhibitors, led us identifying AChE inhibitors in the low micromolar IC_50_ range and with a non-competitive mechanism (Brühlmann et al., 2001). These encouraging preliminary results draw attention to the likelihood of decorating the coumarin skeleton to obtain dual-acting compounds with a particular attention to the substitution pattern branching position 3, 4, and 7 in line with steric prerequisites and synthetic handling.

The introduction of diverse basic functionalities at the 3-position of the 6,7-dimethoxycoumarin furnished promising molecules (Figure 1, general structure **8**), although their activity was biased against MAO B inhibition at the nanomolar level while showing only moderate anti-AChE activity in the low micromolar range (Pisani et al., 2016). Similarly, unbalanced profiles were observed after the design and the screening of a small collection bearing head-or-tail modifications on 4-aminomethyl-7-benzyloxy-2H-chromen-2-one motif (Figure 1, general structure **7**) (Rullo et al., 2019). The SAR indicated a preference for halogens as the *meta*-substituents of the benzyl ring to increase multitargeting activity. Interestingly, during these efforts triple-acting AChE/BChE/MAO B inhibitors were discovered, which could benefit from the additional inhibition of BChE whose increased expression and activity has been documented in chronic AD (Arendt et al., 1992). Upon applying “design-in” approaches (Farina et al., 2015) the molecular framework of coumarin-based multitarget ligands was built by exploiting the ability of the MAO B active site to accommodate 4-unhindered 7-benzyloxy-2*H*-chromen-2-one rings, which were tethered to *N*-benzylaminomethyl basic heads in order to improve AChE inhibition (Figure 1, general structure **9**). By hybridizing the coumarin core with a protonatable group and a nitrate ester, we obtained triple-acting chemotypes able to inhibit AChE and MAO B, and to release low neuroprotective doses of nitric oxide (Pisani et al., 2019). Furthermore, structural variations led to improve the drug-likeness of the hybrid hit compounds, e.g., water solubility (Pisani et al., 2016). To this scope, the entropic penalties introduced by more flexible spacers were mitigated by the xylyl/polymethylene chain to piperidine ring exchange that allowed more favorable p*K*_a_ values thanks to the *N*-benzylpiperidine moiety (Figure 1, general structure **10**), inspired by donepezil. Cell-based assays showed that some of the hits presented low cytotoxicity and increased cell viability in neuroblastoma cell lines insulted by pro-oxidative toxins such as hydrogen peroxide, along with *in vitro* dual target’s inhibition from sub-micromolar to low nanomolar range and outstanding MAO B/A selectivity. Moreover hits **9a** (Farina et al., 2015) (IC_50_ = 10 and 120 nM toward MAO B and AChE, respectively; MAO B/A selectivity >1,000) and **10a** (Pisani et al., 2016) (IC_50_ = 30 nM and 1.0 μM toward MAO B and AChE, respectively; MAO B/A selectivity >90) behaved as CNS-permeant compounds in a BBB-mimicking model without efflux pump liabilities arising from P-glycoprotein interactions. For dual-targeting compound **9a** the earliest X-ray crystallographic structures upon binding mutually to MAO B and AChE have been recently reported (Ekström et al., 2022). As challenging alternative, H/F and OH/CF_2_H bioisosteric mimicry led to a potent, reversible, and drug-like derivative as proved in early-ADME studies (compound **9b**; IC_50_ = 8.2 and 550 nM toward MAO B and AChE, respectively; MAO B/A selectivity >1,200 (Rullo et al., 2022).

## Coumarins as antitumor agents

Coumarin itself or many of its derivatives showed antitumor activity by inhibiting the proliferation of the tumor cells at very low concentration without showing toxicity towards healthy cells. Their antitumor activity is due to (Wu et al., 2020) the inhibition of many signalling pathways such as PI3K/AKT/mTOR (Ma et al., 2020), microtubule polymerization (Madari et al., 2003; Liu et al., 2015) and angiogenesis. They also act on apoptotic proteins (Chuang et al., 2007) and on ROS regulation (Kontogiorgis et al., 2007), and through the inhibition of enzymes such as aromatase (Chen et al., 2004) and estrogen sulfatase (Stanway et al., 2006) both involved in breast cancer progression.

Aromatase (AR) is a multienzymatic complex, composed by cytochrome P450 (CYP19) and a NADPH-cytochrome P450 flavoprotein reductase, and it is localized in the endoplasmic reticulum of the cell.

AR controls the conversion of androgens to estrogens, catalyzing the aromatization of the steroidal enone, present in the A ring, into the corresponding phenolic ring trough the oxidation and the consequent elimination of the C19 methyl group. Many AR inhibitors contain an azole ring able to coordinate the iron ion of the AR heme group by means of their lone pair.

**FIGURE 3:**
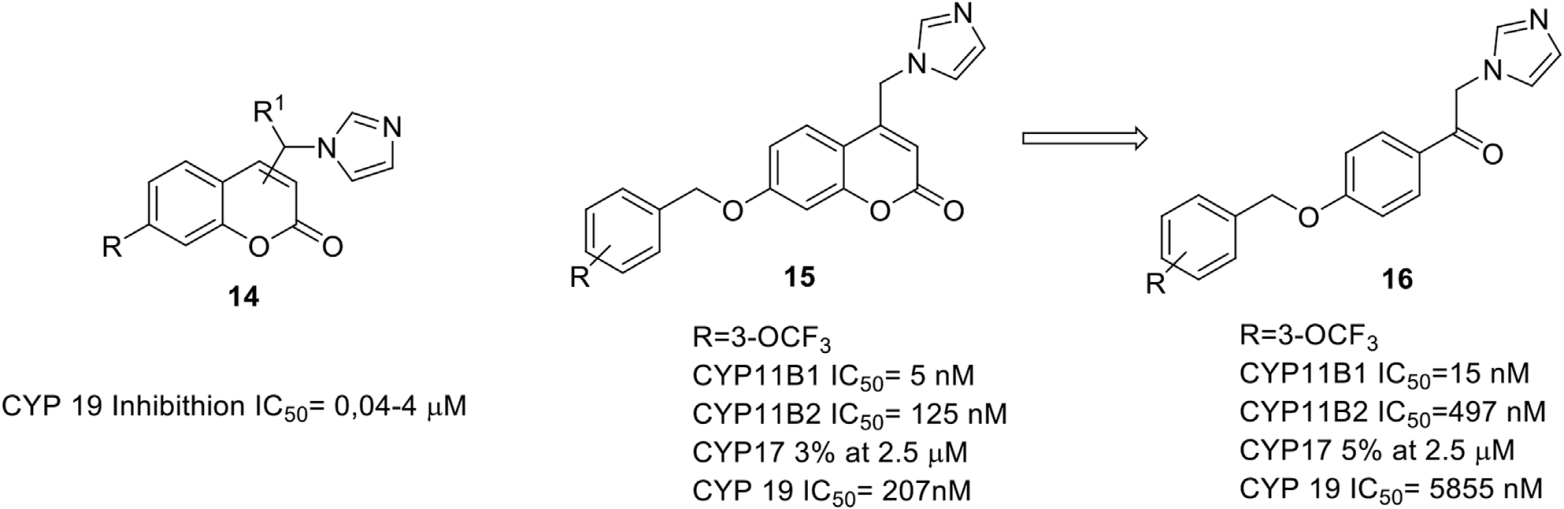
General structure of aromatase inhibitors. (Stefanachi et al., 2011; Stefanachi et al., 2015).

The design of our inhibitors started with the evaluation of the introduction of the appropriate azole substituent on our coumarin core. The best results were obtained by 4-imidazolyl coumarin derivatives (Leonetti et al., 2004; Stefanachi et al., 2011) bearing in 7-position a phenoxy or benzyloxy substituent as illustrated in Figure 3 (**14**).

A three sites interaction model was proposed: 1) the binding driving interaction is the heme iron coordination by the lone pair of the electron rich imidazole, 2) the coumarin ring is positioned perpendicularly to the imidazole ring, and its lactone group establish a key hydrogen bond with S478, 3) the phenoxy and benzyloxy groups fill a hydrophobic pocket. We were able to obtain inhibitory activities in the range of 40–400 nM, comparable with that of fadrozole, in particular for the phenoxy derivatives.

All the synthesized compounds showed selectivity for AR with no activity towards other cytochromes P such as CYP17 (C17,20-lyase), CYP11B1 (steroid 11β-hydroxylase) and CYP11B2 (aldosterone synthase).

We also designed and synthesized acyclic analogs of coumarin derivatives (**16**, Figure 3), and the data showed that the absence of the coumarin ring does decrease the aromatase inhibitory activity, while increasing the potency at CYP11B1 and selectivity over CYP11B2, CYP19, and CYP17 (Stefanachi et al., 2015).

## Conclusion

Herein, we review the main results of the pioneering scientific journey started about 20 years ago under the wise guide of Prof. Angelo Carotti (Bari, Italy) to explore the bioactivity potential of the coumarin core. Several programs on coumarin derivatives as single-targeting MAO, followed by AChE inhibitors as well as MTDLs, have been carried out focusing on the potential pharmacological treatment of neurodegenerative syndromes. Moreover, some coumarin derivatives also showed aromatase inhibitory activity and, therefore, have been developed as potential therapeutic agents for the treatment of breast cancer.Cao et al., 2016, Pisani et al., 2013.

## Author's note

**Dedicated to prof. Angelo Carotti in the occasion of his 76th birthday.**
